# Lactate and lactylation in cancer: drivers of immune suppression and microenvironmental reprogramming

**DOI:** 10.1186/s40164-025-00719-3

**Published:** 2025-10-28

**Authors:** Xiaoyu Ji, Limin Xia

**Affiliations:** Department of Gastroenterology, Institute of Liver and Gastrointestinal Diseases, Hubei Key Laboratory of Hepato-Pancreato-Biliary Diseases, Tongji Hospital, Tongji Medical College, State Key Laboratory for Diagnosis and Treatment of Severe Zoonotic Infectious Diseases, Huazhong University of Science and Technology, Wuhan, 430030 Hubei Province China

**Keywords:** Lactate, Lactylation, Tumor microenvironment, Tumor progression, Targeting therapy

## Abstract

Lactate, a key metabolite of the Warburg effect, plays a central role in shaping multiple hallmarks of cancer. Through lactate shuttling and engagement with specific receptors, it activates downstream signaling pathways that remodel the tumor microenvironment (TME) and facilitate tumor progression. More recently, lysine lactylation—an emerging post-translational modification derived from lactate—has been identified as a crucial epigenetic mechanism that links altered tumor metabolism with transcriptional regulation. Lactylation has been implicated in promoting tumor proliferation, metastasis, stemness maintenance, immune evasion, and therapeutic resistance across various cancer types. Both tumor and immune cells undergo lactylation, which modulates gene expression and contributes to the immunosuppressive landscape of the TME. Targeting lactate production and transport has shown promise in suppressing tumor growth and enhancing immunotherapeutic efficacy. In this review, we comprehensively discuss the functional roles and underlying mechanisms of lactate and lactylation in cancer progression, with a particular focus on their impact within the TME. We also highlight recent advances in targeting these metabolic processes as potential therapeutic strategies, aiming to provide new perspectives for improving cancer treatment outcomes.

## Background

To support their proliferation, metastasis, and survival, tumor cells undergo extensive metabolic reprogramming. Tumor cells preferentially utilize glycolysis over oxidative phosphorylation to fulfill their energy demands, even under aerobic conditions—a phenomenon known as the Warburg effect [1–3]. This metabolic reprogramming allows cancer cells not only to generate adenosine triphosphate (ATP) but also to produce essential metabolic intermediates that support cell growth, proliferation, and biosynthetic processes [4, 5]. Lactate is an important metabolite of the Warburg effect. The level of lactate is significantly increased in many types of tumors and indicates a poor prognosis [6, 7].

The accumulation of large amounts of lactate promotes tumor proliferation, metastasis, and treatment resistance and affects the efficiency of tumor immune treatment [8]. Lactate exerts its critical roles in the following ways: (1) as energy support to meet the needs of tumor growth (2) lactate participates in signal transduction by binding to its receptors to promote tumor survival and progression (3) a large amount of lactate in tumor cells is transported to the microenvironment through monocarboxylate transporters (MCTs), which alleviates the cell damage caused by lactate accumulation. Meanwhile, it contributes to the acidification of the tumor microenvironment (TME) and reshapes the immune landscape [9–11].

Metabolism reprogramming and epigenetic modifications both are hallmarks of cancer [12]. Lactylation, a newly identified post-transcriptional modification, was firstly discovered in 2019 and closely links these two features together [13]. The discovery of lactylation shed light on the understanding of epigenetic regulation and cell metabolism. Lactylation is widely occurred in lysine residues of both histone and non-histone proteins, which regulates genes expression involved in cancer development [14–16].

In this review, we summarize the roles and underlying mechanisms of lactate and lactylation in malignant progression, with a particular focus on the tumor microenvironment and immune evasion. We also highlight key findings regarding their potential as therapeutic targets in cancer treatment. A deeper understanding of how lactate and lactylation regulate tumor progression and the immune microenvironment may provide a theoretical foundation for developing novel cancer treatment strategies.

### Metabolic pathways drive lactate production in tumor cells

The high glycolytic activity of tumor cells results in excessive lactate production, making it the primary source of lactate. This glycolytic process is initiated by the uptake of glucose from the extracellular space through glucose transporters. Glucose is then converted into glucose-6-phosphate, fructose-1,6-bisphosphate, and pyruvate through a series of enzymatic reactions. Finally, pyruvate is converted into lactate through the catalysis of lactate dehydrogenase (LDH), and ATP is generated in the process for energy supply [17, 18].

**Fig. 1:**
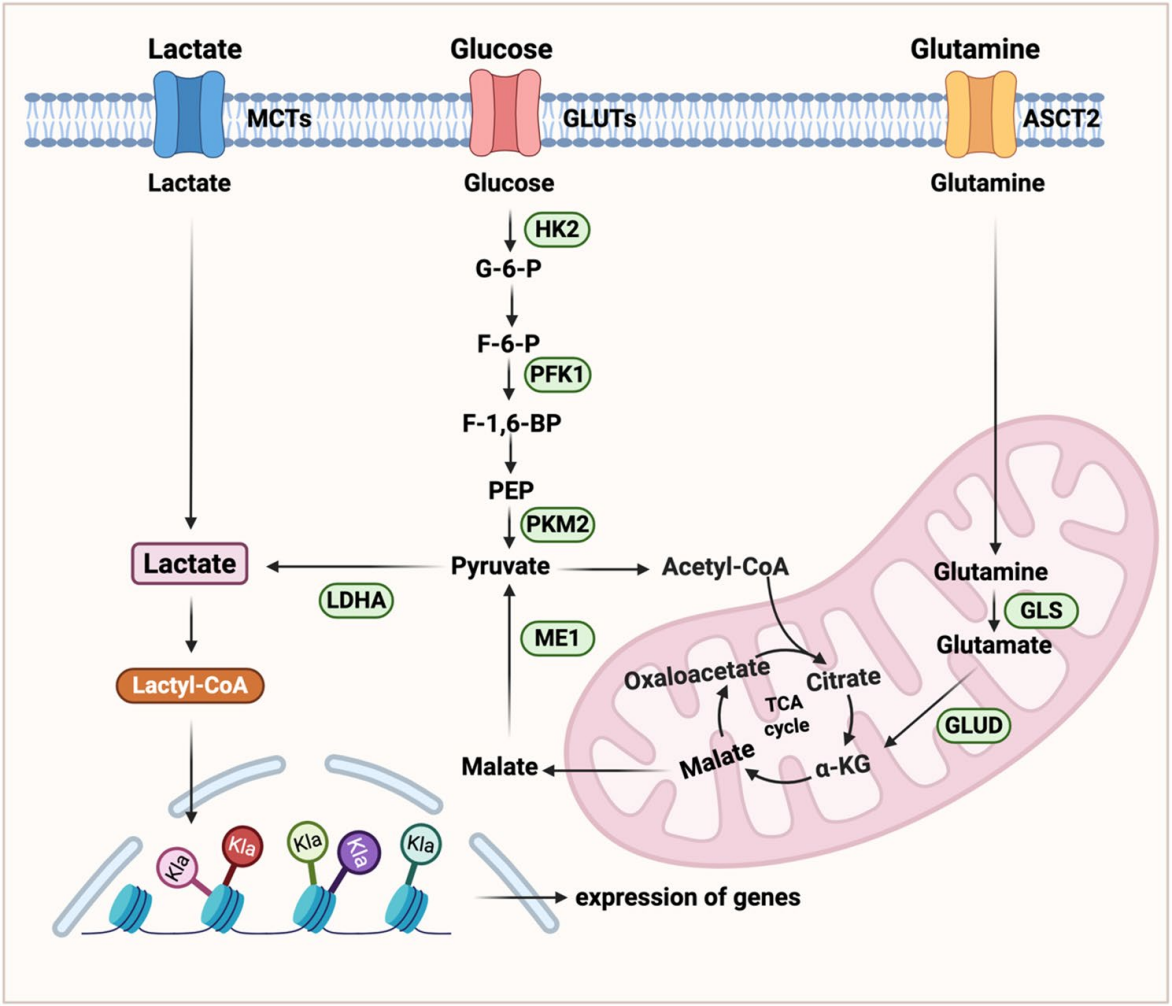
Lactate metabolism in tumor cells. There are three main sources of lactate in tumor cells: (1) Glucose is transported into cells through GLUTs, and glucose is converted into pyruvate through a series of enzymatic reactions, and finally lactate is generated under the catalysis of LDHA; (2) Lactate in the tumor microenvironment is directly transported into tumor cells through MCTs; (3) Glutamine is converted into α-KG in cells and enters the TCA cycle to supplement pyruvate, and finally converted to lactate. Importantly, lactate is converted into lactyl-CoA, which mediates the lactylation of histones and non-histones to regulate gene expression

Lactate can also be converted from glutamine in a series of enzymatic reactions [19]. Glutamine is transported into the cell through amino acid transporter type 2 (ASCT2) on the cell membrane [20]. In the mitochondria, glutamine generates glutamate under the action of glutaminase (GLS). Glutamate is converted into α-ketoglutarate by glutamate dehydrogenase (GLUD), and α-KG is converted into malate in the mitochondria through part of tricarboxylic acid (TCA) cycle reactions. In the cytosol, malate is converted to pyruvate by malic enzyme 1 (ME1), which is subsequently reduced to lactate [21]. This metabolic pathway is frequently upregulated in tumor cells to provide critical metabolites such as pyruvate, lactate, and nicotinamide adenine dinucleotide phosphate (reduced form, NADPH), thereby supporting anabolic growth and redox homeostasis (Fig. 1).

### MCTs mediate lactate shuttling and maintain tumor metabolic networks

Monocarboxylate transporters mediate the bidirectional shuttling of lactate between tumor cells and the TME. These transporters are encoded by the solute carrier family 16 (SLC16), which comprises 14 members (SLC16A1-SLC16A14), and are critically involved in nutrient transport, cellular metabolism, and pH homeostasis [22–24]. Among them, MCT1–MCT4 are the most extensively studied proton-linked transporters. In addition to facilitating lactate transport across the plasma membrane, they are also responsible for the transmembrane movement of pyruvate, ketone bodies (e.g., acetoacetate and β-hydroxybutyrate), and other essential monocarboxylates [25, 26]. The transport cycle begins with the binding of a proton to the MCT, which promotes subsequent lactate binding. This triggers a conformational change in the transporter, enabling the co-transport of lactate and protons across the membrane. Notably, the release of lactate precedes the dissociation of the proton on the opposite side of the membrane.

MCT1 (SLC16A1) is ubiquitously expressed across various tissues, whereas MCT2 (SLC16A7) shows predominant expression in the liver, kidney, testis, and brain. MCT4 (SLC16A3) is mainly localized in highly glycolytic tissues such as white skeletal muscle, astrocytes, and white blood cells. The expression of MCT1, MCT2, and MCT4 is markedly upregulated in a wide range of tumors and is closely associated with enhanced glycolytic activity [27]. MCTs-mediated lactate transport between glycolytic and oxidative tumor cells plays a critical role in metabolic coupling and intercellular communication within the tumor microenvironment.

### Lactate functions as a signaling molecule to regulate tumor behavior

Lactate functions as a signaling molecule by binding to its receptors, thereby regulating diverse aspects of tumor biology, including immune modulation, metabolic reprogramming, and tumor progression. The most well-characterized lactate receptors include G protein-coupled receptor 81 (GPR81), GPR132, and GPR65.

GPR81 was initially identified as a lactate receptor predominantly expressed on the surface of adipocytes and muscle cells [28]. Subsequent studies have demonstrated that GPR81 expression is upregulated in a variety of malignancies, including colon, breast, liver, lung, pancreatic, cervical, and salivary gland cancers. Notably, GPR81 is expressed not only on the surface of tumor cells but also on non-malignant cells within the TME, suggesting a broader role in tumor-host interactions [29]. Lactate generated by tumor cells is exported through MCTs and engages GPR81 on the same cell surface to initiate autocrine signaling cascades. In parallel, lactate released by stromal or immune cells in the tumor microenvironment can activate GPR81 on neighboring cancer cells in a paracrine fashion. Conversely, lactate originating from tumor cells may also signal to adjacent non-malignant cells via GPR81, highlighting a bidirectional lactate-GPR81 axis within the tumor niche. When lactate interacts with GPR81, it initiates a Gi protein-mediated signaling pathway that downregulates adenylate cyclase function, thereby diminishing intracellular cyclic adenosine monophosphate (cAMP) levels. This signaling cascade contributes to metabolic reprogramming, immune evasion, and enhanced tumor cell survival in cancer [30, 31].

GPR132, also known as G2A, a member of the G protein-coupled receptor family, has recently been characterized as a sensor for extracellular lactate. GPR132 is primarily expressed on the surface of immune cells and plays a critical role in regulating their proliferation, migration, and differentiation [32, 33]. For instance, lactate can bind to GPR132 on natural killer (NK) cells, suppressing the expression of interferon-γ (IFN-γ) and granzyme B (GZMB), thereby facilitating tumor progression [32]. Similarly, lactate derived from tumor cells can engage GPR132 on macrophages, promoting their polarization toward the M2 phenotype, which is associated with immunosuppression and tumor-supportive functions [33].

GPR65, also known as TDAG8, is a proton-sensitive GPCR that senses the acidic TME and transduces lactate- and acidosis-derived signals to regulate tumor and immune cell function [34, 35]. In tumor cells, GPR65 activation promotes survival and proliferation by engaging cAMP/ protein kinase A (PKA) and MAPK signaling, thereby enhancing adaptability to acidic stress and driving tumor progression [36]. High GPR65 expression correlates with poor prognosis in several solid tumors [37]. Notably, GPR65 expression also influences immunotherapy outcomes: in a mouse model of B-cell acute lymphoblastic leukemia, low GPR65 expression conferred resistance to CD19 + CAR T therapy, partly by remodeling tumor–macrophage interactions. GPR65 deficiency increased vascular endothelial growth factor A (VEGFA) production in tumor cells, which expanded macrophages and skewed them toward an M2-like immunosuppressive phenotype [38]. Beyond tumor cells, GPR65 is highly expressed on immune populations such as tumor-associated macrophages (TAMs) [39, 40]. In obesity-driven cancer models, oleic acid accumulation creates an acidic milieu that is sensed by TAMs via GPR65, promoting their polarization toward a protumorigenic state and accelerating tumor growth [39]. In gliomas, GPR65 serves as the primary lactate receptor on TAMs, where it activates the cAMP/PKA/CREB pathway, induces high mobility group box 1 (HMGB1) secretion, and fosters glioma progression. Pharmacologic inhibition of GPR65 has demonstrated anti-glioma potential in preclinical models [35]. Collectively, GPR65 emerges as a key regulator of tumor–immune interactions and a promising therapeutic target in the acidic TME.

## Lactate reprograms tumor cell metabolism and drives malignant progression

**Fig. 2:**
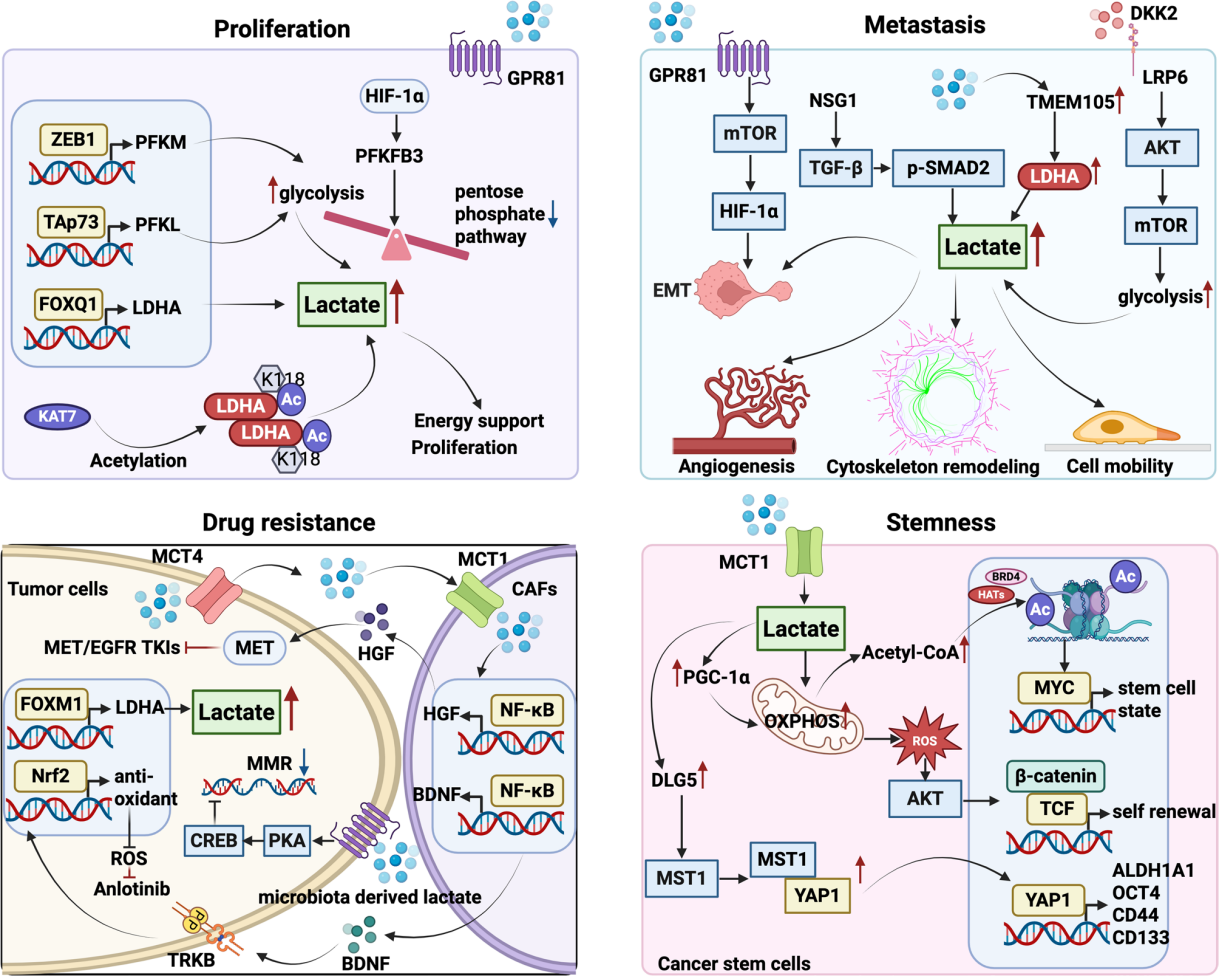
Lactate drives tumor cell malignancy. Lactate promotes tumor growth, facilitates metastasis, sustains stemness, and increases resistance to chemotherapy

For maintaining continuous growth and survival, tumor cells have a significantly increased demand for energy. As a key metabolite produced during glycolysis, lactate not only behaves as an energy substrate to support tumor cell metabolism, but also promotes tumor progression in many ways. Accumulating evidence supports that lactate can enhance the proliferation ability, metastatic potential and angiogenesis level of tumor cells, while also participating in maintaining stemness and mediating their resistance to anticancer treatment, further promoting the formation of a malignant phenotype (Fig. 2).

### Proliferation

The role of lactate in promoting tumor proliferation has been widely studied. Lactate is abundant in rapidly dividing cells due to the need for elevated glucose metabolism to support proliferation. Lactate is responsible for promoting the proliferation of various tumors such as hepatocellular carcinoma, breast cancer, and lung adenocarcinoma [41–43]. For example, zinc finger E-box-binding homeobox 1 (ZEB1) promotes hepatocellular carcinoma (HCC) proliferation by activating the expression of muscle isoform of phosphofructokinase-1 (PFKM), a key enzyme in the Warburg effect, leading to enhanced glycolysis and elevated lactate levels. Similarly, transactivation domain-containing p73 (TAp73) accelerates tumor proliferation by upregulating the expression of phosphofructokinase-1, liver type (PFKL), another key enzyme in glycolysis, which enhances glucose consumption and lactate production [44, 45]. In addition, lactate enhances the pentose phosphate pathway by upregulating the activity of glucose-6-phosphate dehydrogenase (G6PD), thereby supporting redox homeostasis and promoting the proliferation of breast cancer cells [46].

Moreover, LDHA-mediated lactate production is a central driver of tumor proliferation. Tumor cells enhance lactate accumulation and sustain proliferative capacity by upregulating LDHA expression and enzymatic activity [47–49]. In pancreatic cancer, for instance, the transcription factor Forkhead box Q1 (FOXQ1) directly activates LDHA transcription, thereby increasing lactate output and fueling tumor growth [48]. Beyond transcriptional regulation, post-translational modification further amplifies LDHA activity. Acetylation at lysine 118, catalyzed by lysine acetyltransferases 7 (KAT7), enhances both LDHA enzymatic function and protein stability, ultimately promoting tumor proliferation [49].

### Metastasis

Metastasis, defined by the spread and colonization of tumor cells at distant anatomical sites, remains the primary driver of cancer-associated death [50]. Lactate, a key byproduct of glycolysis, facilitates metastasis by promoting epithelial–mesenchymal transition (EMT), angiogenesis, and other pro-metastatic processes.

Enhanced lactate production within tumor cells plays a pivotal role in driving metastasis. In breast cancer, lactate upregulates transmembrane protein 105 (TMEM105), which in turn promotes glycolysis and LDHA-mediated lactate generation, establishing a positive feedback loop that facilitates liver metastasis [51]. In prostate cancer, pharmacological inhibition of LDHA with FX11 markedly suppresses lactate-driven tumor cell migration and angiogenesis [52]. Similarly, in colorectal cancer (CRC), tumor-secreted Dickkopf-associated protein 2 (DKK2) accelerates lactate production by enhancing glycolysis. Mechanistically, DKK2 interacts with lipoprotein receptor-related protein 6 (LRP6) to activate the PI3K/Akt/mTOR pathway, thereby fueling lactate production and promoting angiogenesis [53]. In esophageal squamous cell carcinoma (ESCC), neuronal vesicle trafficking-associated protein 1 (NSG1) amplifies glycolysis and lactate production through activation of the TGF-β/p-SMAD2 axis, which drives epithelial–mesenchymal transition (EMT) and accelerates tumor progression [54].

Beyond enhancing lactate production, lactate also promotes migration, invasion, and acquisition of a mesenchymal-like phenotype by activating downstream signaling pathways [55, 56]. For instance, lactate–GPR81 signaling triggers the mTORC1/HIF-1α pathway, thereby inducing EMT and facilitating CRC metastasis [57]. Lactate promotes cervical cancer cell invasion and metastasis by remodeling the cytoskeleton through promoting the entry of β-catenin into the nucleus and the exit of fascin from the nucleus [58].

### Drug resistance

Elevated lactate levels have been observed in a variety of therapy-resistant tumors. The preceding summary suggests that lactate has been implicated in promoting chemoresistance across multiple cancer types. Drug-resistant cancer cells frequently exhibit metabolic rewiring, marked by a glycolytic shift and elevated lactate output. Notably, inhibition of lactate synthesis has been shown to restore sensitivity to both chemotherapy and immunotherapy. Collectively, these findings highlight a critical role for lactate in mediating tumor drug resistance [59–62].

LDHA-mediated lactate production has emerged as a critical driver of tumor drug resistance. In breast cancer, Forkhead box M1 (FOXM1) has been identified as a biomarker of resistance to PI3Kα inhibitors. Mechanistically, overexpression of FOXM1 enhances LDHA-mediated lactate production, thereby promoting resistance to PI3Kα inhibitors. Importantly, the combination of PI3Kα inhibitors with the hormone therapy drug tamoxifen significantly reduces LDHA expression and lactate levels in taselisib-resistant xenograft models, effectively overcoming resistance [63, 64]. Similarly, lactate accumulation also contributes to chemoresistance in other tumor contexts by modulating downstream signaling pathways. In cisplatin-resistant non-small cell lung cancer (NSCLC) cells, elevated lactate levels suppress the expression of Forkhead box O3 (FOXO3) through YTH N6-methyladenosine RNA binding protein 2 (YTHDF2)-mediated N6-methyladenosine (m6A) modification. This epigenetic regulation enhances the proliferative capacity and tumor growth of cisplatin-resistant NSCLC cells both in vitro and in vivo [62].

Beyond its autocrine effects on tumor cells, lactate also orchestrates intercellular communication within the tumor microenvironment, notably by reprogramming cancer-associated fibroblasts (CAFs) to foster therapeutic resistance [59, 65]. For instance, the multikinase inhibitor Anlotinib suppresses gastric cancer (GC) cell proliferation by inducing apoptosis and arresting the cell cycle at the G2/M phase. However, this antitumor efficacy is markedly reduced when GC cells are co-cultured with CAFs, underscoring the protective role of the stromal compartment. Mechanistically, lactate secreted by GC cells promotes drug treatment resistance by promoting NF-κB activation and brain-derived neurotrophic factor (BDNF) secretion in CAFs, and BDNF, in turn, acts on GC cells to promote Anlotinib resistance [65].

In addition, lactate produced by microbiota similarly promote tumor drug resistance. Lactate produced by the tumor-resident bacterium Lactobacillus in cervical cancer promotes therapeutic resistance by regulating tumor cell metabolism [66]. Gut microbiota C. tropicalis promotes chemoresistance to oxaliplatin in CRC by promoting tumor cell glycolysis and lactate production to inhibit the mismatch repair system [67].

### Cancer cell stemness

Cancer cell stemness represents a central driver of cancer development. Cancer stem cells (CSCs) possess hallmark properties, including strong tumor-initiating potential, self-renewal, and multilineage differentiation within the tumor [68]. Emerging evidence indicates that lactate sustains tumor stemness and accelerates progression by reprogramming CSCs metabolic signatures and regulating gene expression.

In CRC, non-CSCs and CSCs display distinct metabolic phenotypes: non-CSCs preferentially rely on glycolysis and generate abundant lactate, whereas CSCs depend more on oxidative phosphorylation (OXPHOS). Lactate derived from non-CSCs enhances CSC organoid formation and tumor-initiating capacity by activating OXPHOS and the AKT–Wnt/β-catenin pathway. Notably, CSCs in CRC can be further classified into hypoxic and normoxic subsets. Normoxic CSCs, enriched in vascularized regions, exhibit a greater propensity for dissemination and metastasis. In this context, microenvironmental lactate promotes peroxisome proliferator-activated receptor-γ coactivator-1α (PGC-1α)–mediated OXPHOS, thereby facilitating metastatic spread and tumor progression [69, 70].

In addition to its metabolic activity, lactate promotes CSCs enrichment by modulating gene expression networks that inhibit differentiation and induce dedifferentiation. Mechanistically, lactate promotes histone acetylation and enhances MYC activation through increased chromatin accessibility, reinforcing tumor stemness [71]. Similar mechanisms are observed in other cancers, such as OSCC, where CAFs–derived lactate augments CSCs stemness [72].

### Lactate shapes immune cell function to promote immunosuppression

As a hallmark of the Warburg effect, tumors generate excessive lactate, which contributes to the development of a hypoxic and acidified tumor microenvironment. Beyond its metabolic role, lactate functions as a pivotal signaling molecule, mediating dynamic interactions between malignant cells and the surrounding stromal and immune compartments. Through regulating immune checkpoint expression, orchestrating the recruitment and polarization of immunosuppressive cell populations, and dampening the cytolytic functions of T cells and NK cells, lactate actively drives immune evasion and tumor progression.

### Lactate regulates immune checkpoint expression

Programmed death-ligand 1 (PD-L1) has an irreplaceable role in tumor immune escape and the establishment of an immunosuppressive tumor microenvironment. Lactate has been shown to upregulate PD-L1 expression on the surface of tumor cells. Mechanistically, lactate activates the GPR81, leading to reduced intracellular cAMP levels and decreased PKA activity. This cascade promotes the activation of the transcriptional co-activator transcriptional coactivator with PDZ-binding motif (TAZ), which, in cooperation with the transcription factor TEA domain family member (TEAD), drives PD-L1 expression in lung cancer cells [73]. Similarly, IL-6-driven lactate production upregulates PD-L1 expression on uveal melanoma cells through activation of the GPR81-cAMP-PKA signaling cascade, thereby promoting immune evasion and facilitating tumor progression [74].

### Lactate reprograms cancer-associated fibroblasts

Lactate produced by tumor cells can be imported by CAFs within the tumor microenvironment. For example, in lung cancer, tumor-derived lactate promotes the nuclear translocation of nucleolar and spindle associated protein 1 (NUSAP1) and the transcriptional activity of DESMIN in CAFs, thereby driving their activation. Activated CAFs subsequently recruit TAMs through IL-8 secretion, contributing to tumor progression [75]. LDHA enhances glycolysis and lactate production in pancreatic ductal adenocarcinoma (PDAC) cells, whereas CAFs utilize tumor-derived lactate via MCT1 as an energy source to sustain their proliferation. Moreover, lactate stimulates CAFs to secrete IL-6, thereby fostering an immunosuppressive microenvironment that facilitates tumor progression [76].

Conversely, lactate released by CAFs is readily taken up by tumor cells and fuels their metabolic demands. In prostate cancer, CAFs-derived lactate induces lipid metabolic reprogramming in tumor cells, leading to increased lipid droplet accumulation and providing acetyl-CoA for histone acetylation, thus enhancing tumor invasiveness [77].

### Lactate shapes immune cell phenotypes

**Fig. 3:**
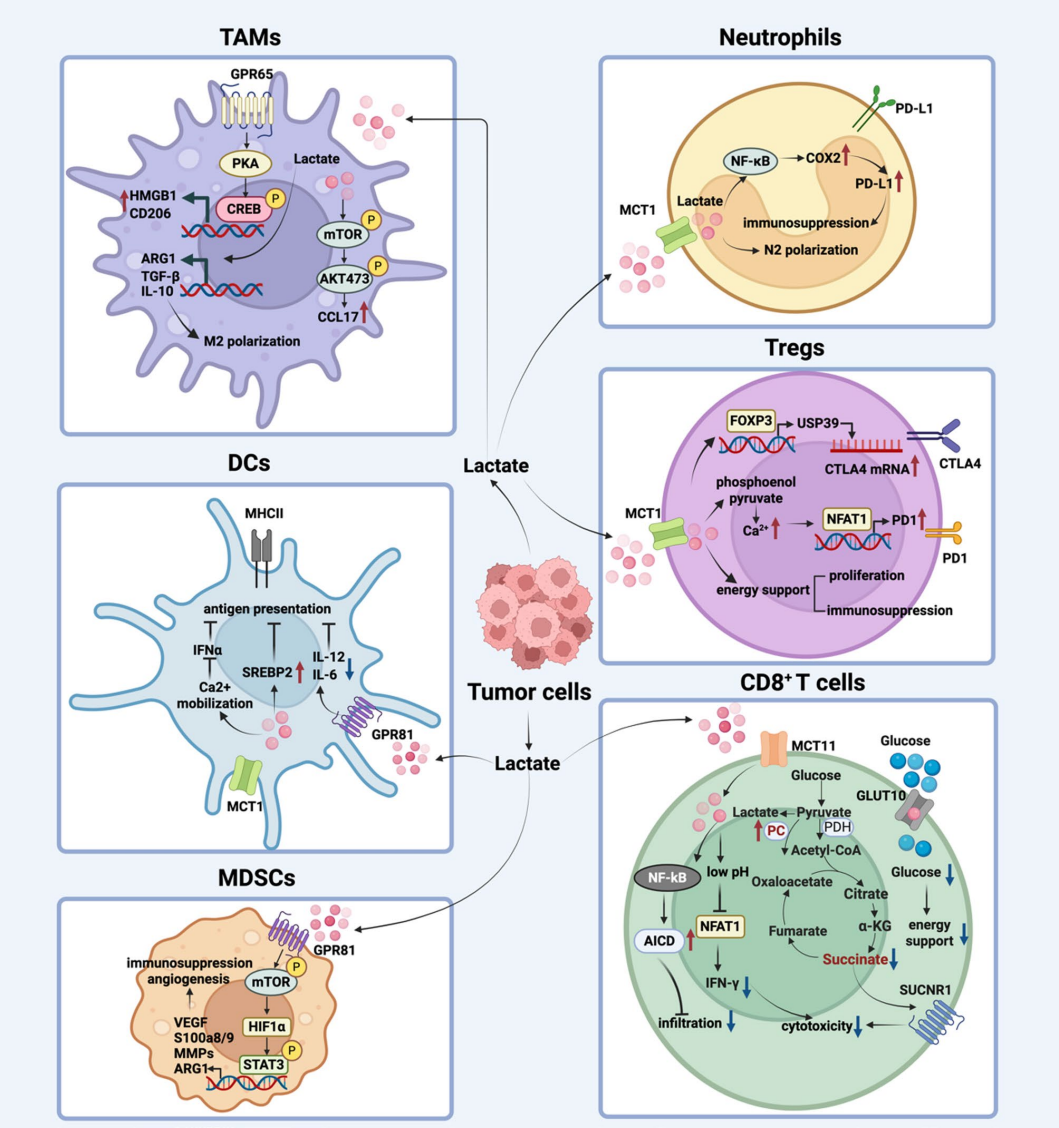
The effect of lactate on immune cells in the TME. Tumor cells secrete a large amount of lactate into the TME. On the one hand, lactate inhibits the antigen presentation function of dendritic cells and the tumor killing effect of CD8⁺ T cells. On the other hand, lactate enhances the immunosuppressive function of Tregs, MDSCs, TAMs and neutrophils

Tumor cells release large quantities of lactate into the TME, accompanied by an accumulation of H^+^ that leads to increased acidity of TME. This acidic environment impairs the function of anti-tumor immune cells. Moreover, lactate enhances the infiltration and immunosuppressive activity of regulatory immune cells while suppressing the survival and cytotoxic function of anti-tumor immune cells, thereby contributing to tumor progression (Fig. 3).

### Regulatory T cells (Tregs)

Tregs, characterized by Forkhead box P3 (Foxp3) expression, are key players in establishing an immunosuppressive tumor microenvironment. Growing evidence highlights lactate as a key regulator of both the expansion and immunosuppressive function of Tregs. Within the tumor microenvironment, lactate shapes antitumor immunity by modulating Treg infiltration and activity [78–80].

On one hand, lactate drives tumor cells and CAFs to secrete chemokines that promote Treg infiltration into the TME. In gastric cancer, tumor-derived lactate engages GPR81, initiating the nuclear translocation of phosphorylated p65 and activating C-X3-C motif chemokine ligand 1 (CX3CL1) transcription. This chemokine facilitates the infiltration of Tregs, which in turn suppress CD8⁺ T cell cytotoxicity and promote tumor progression [81]. Moreover, lactate uptake by fibroblasts facilitates their transformation into inflammatory CAFs (iCAFs), which in turn promote Tregs infiltration through the hypoxia-inducible factor 1-alpha (HIF1α)/ C-X-C motif chemokine ligand 12 (CXCL12) axis [82].

On the other hand, Tregs exploit lactate within the TME to enhance their immunosuppressive capacity by both supporting metabolic demands and modulating gene expression. CAFs-derived lactate influences T cell differentiation to further promote tumor progression. It reduces the proportion of anti-tumor Th1 cells while enhancing NF-κB activation and Foxp3 expression in naïve T cells, thereby driving their differentiation into Tregs [83]. Tregs acquire lactate through MCT1 on their surface, which facilitates intracellular signaling cascades. Elevated lactate uptake enhances Foxp3 expression, thereby inducing ubiquitin-specific peptidase 39 (USP39), a factor essential for RNA splicing events that sustain cytotoxic T-lymphocyte associated protein 4 (CTLA4) expression. This pathway reinforces Tregs-mediated immune suppression and undermines the efficacy of CTLA4-targeted therapies [84].

Lactate also impacts responses to anti-PD-1 immunotherapy by modulating programmed death-1 (PD-1) expression in a cell-type-specific manner. In Tregs, lactate promotes nuclear translocation of nuclear factor of activated T cells 1 (NFAT1), leading to PD-1 upregulation. In contrast, it suppresses PD-1 expression on CD8⁺ T cells. This differential regulation disrupts the balance between effector and suppressive T cell populations, ultimately limiting the therapeutic benefit of PD-1 blockade [85].

### Tumor-associated macrophages (TAMs)

TAMs are pivotal drivers of tumor progression, immune suppression, and resistance to immunotherapy. Their protumorigenic functions are mediated in part through the secretion of immunosuppressive cytokines such as IL-10, arginase 1 (ARG1), and TGF-β. Lactate has emerged as a central regulator of TAM biology, orchestrating M2 polarization and sustaining their immunosuppressive phenotype [86–88].

In the TME, lactate produced and secreted by tumor cells exerts profound regulatory effects on TAMs. For example, in STK11/LKB1-mutant lung adenocarcinoma, tumor cells export lactate via MCT4, which promotes M2-like polarization of TAMs while impairing T cell function. Silencing MCT4 partially reverses this phenotype and enhances the efficacy of immune checkpoint blockade (ICB) [89]. In addition, lactate participates in bidirectional crosstalk between tumor cells and TAMs. In breast cancer, TAMs transfer the myeloid-specific long non-coding RNA, HIF-1α-stabilizing long noncoding RNA (HISLA), to tumor cells via extracellular vesicles, augmenting glycolysis and apoptosis resistance; conversely, tumor-derived lactate upregulates HISLA in TAMs, establishing a feedforward loop that reinforces tumor progression [90].

At the level of lactate-mediated signaling, tumor-derived lactate activates multiple pathways in immune cells that subsequently influence tumor behavior. In pituitary adenoma, lactate activates the mTOR pathway in TAMs, leading to increased secretion of C-C motif chemokine ligand 17 (CCL17), which binds to C-C motif chemokine receptor 4 (CCR4) on tumor cells and promotes invasion [91]. Similarly, in CRC, lactate accumulation mediated by PCSK9 and SETDB1 facilitates M2-like polarization of macrophages through upregulation of markers such as CD206, TGF-β, and ARG1 in TAMs, accelerating tumor progression [92, 93].

Lactate also signals through cell surface receptors. For instance, lactate binds to GPR65 on the surface of TAMs, activating the cAMP/PKA/CREB signaling axis and triggering the release of HMGB1. This lactate-driven signaling cascade promotes tumor cell proliferation, migration, invasion, and EMT, further contributing to an aggressive tumor phenotype [35, 94].

Besides, reactive oxygen species (ROS) in the TME critically influence macrophage polarization and immunosuppressive programming [95]. Notably, lactate also modulates TAM biology through ROS signaling. Lactate derived from HCC and PDAC cells has been shown to elevate ROS levels in macrophages, thereby driving their polarization toward an M2 phenotype, whereas treatment with the ROS scavenger NAC effectively attenuates this lactate-induced effect [96]. Consistently, lactate exposure markedly increases ROS accumulation in THP-1–derived macrophages and promotes NLR family pyrin domain containing 3 (NLRP3) inflammasome activation in a ROS-dependent manner [97]. Together, these studies highlight a functional link between lactate and ROS in shaping TAMs biology; however, the precise mechanisms underlying this interplay remain incompletely understood and warrant further investigation.

Collectively, these observations position lactate-driven TAMs reprogramming as a pivotal mechanism shaping tumor progression and immune escape, offering new opportunities for therapeutic intervention within the TME.

### Dendritic cells (DCs)

Lactate in the tumor microenvironment has been identified as a potent suppressor of DCs function. It not only impairs the antigen-presenting capacity of DCs but also weakens their ability to mediate T cell cytotoxicity. In vitro, lactate-treated DCs exhibit reduced antigen presentation and diminished cross-presentation capacity. Correspondingly, in vivo studies demonstrate that the antitumor efficacy of lactate-exposed DCs is significantly compromised [98, 99].

In the tumor microenvironment of breast cancer, tumor cells produce and secrete lactate, which is subsequently sensed and taken up by DCs, leading to profound immunoregulatory effects. Tumor-derived lactate binds to GPR81 on tumor-infiltrating conventional DCs, resulting in downregulation of major histocompatibility complex class II (MHCII) expression and suppression of proinflammatory cytokine production. This signaling cascade impairs DCs-mediated priming of CD8⁺ T cells and diminishes their cytotoxic activity [31, 100].

In addition to receptor-mediated signaling, lactate uptake through MCT1 contributes to metabolic reprogramming in plasmacytoid DCs (pDCs). Lactate transport enhances tryptophan metabolism and kynurenine production, which promotes Tregs expansion and fosters an immunosuppressive tumor microenvironment [100].

Furthermore, lactate influences DCs differentiation by modulating intracellular metabolic pathways. Specifically, lactate activates sterol regulatory element-binding protein 2 (SREBP2), facilitating the conversion of immunostimulatory conventional DCs into immunoregulatory CD63⁺ mature regulatory DCs (mregDCs). This metabolic reprogramming inhibits antigen cross-presentation and promotes Treg differentiation, further exacerbating immune suppression [101].

### Neutrophils

Neutrophils are a major component of the immunosuppressive tumor microenvironment, and their increased infiltration is often associated with poor clinical outcomes across multiple cancer types [102, 103].

Lactate contributes to tumor progression and chemoresistance by enhancing the immunosuppressive functions of neutrophils. For instance, lactate is transported into neutrophils via MCT1, where it mediates metabolic and signaling changes that enhance immunosuppressive activity. In HCC, tumor-secreted lactate activates the NF-κB/cyclooxygenase-2 (COX2) pathway in neutrophils, leading to upregulation of PD-L1 expression. This suppresses T cell activation and reduces antitumor immunity, limiting the efficacy of the first-line therapeutic agent Lenvatinib. Pharmacological inhibition of COX2 with celecoxib restores T cell function and improves Lenvatinib efficacy [104].

Furthermore, lactate mediates reciprocal communication between tumor cells and neutrophils. Tumor-secreted lactate promotes neutrophil polarization toward the immunosuppressive N2 phenotype in CRC. These N2 neutrophils transfer the transcription factor spi-1 proto-oncogene (SPI1) to tumor cells via extracellular vesicles, thereby enhancing tumor glycolysis and lactate production, and accelerating tumor progression [105].

### Myeloid derived suppressive cells (MDSCs)

MDSCs are a heterogeneous population of immature myeloid cells that accumulate within the tumor microenvironment and serve as critical regulators of immunosuppression. They impair the cytotoxic functions of T cells and NK cells and contribute to tumor progression through additional mechanisms such as promoting angiogenesis [106]. Lactate also facilitates tumor progression by modulating the infiltration and activity of MDSCs.

Tumor-derived lactate enhances the recruitment and accumulation of MDSCs in the TME, promoting an immunosuppressive milieu that supports tumor progression. Specifically, lactate enhances the production of C-C motif chemokine ligand 2 (CCL2) and C-C motif chemokine ligand 7 (CCL7) by tumor cells in CRC, thereby driving the recruitment of CCR2⁺ polymorphonuclear MDSCs (PMN-MDSCs). These immunosuppressive cells impair CD8⁺ T cell–mediated antitumor responses and support CRC development [107]. Similarly, MCT4-driven lactate efflux has been shown to promote MDSCs infiltration into the TME, thereby reinforcing tumor progression and diminishing responses to immunotherapy [108].

The lactate–GPR81 signaling pathway enhances the immunosuppressive functions of MDSCs within the TME, contributing to tumor progression and therapy resistance. In PDAC, radiotherapy enhances lactate production through amplification of the Warburg effect. This excess lactate engages GPR81 on MDSCs, activating the mTOR-HIF1α-STAT3 signaling axis. Activation of this pathway induces the expression of immunosuppressive mediators, including S100 calcium-binding protein A9 (S100A9) and matrix metalloproteinases (MMPs), which collectively suppress cytotoxic T cell-mediated antitumor immunity and contribute to radiotherapy resistance [109].

ROS are critical for preserving the immature state of MDSCs and enabling their immunosuppressive activity. Emerging evidence indicates that lactate enhances the immunosuppressive capacity of MDSCs through ROS regulation [95, 110]. In vitro, lactate exposure elevates ROS levels in MDSCs and strengthens their ability to suppress CD4⁺ T cell proliferation. Mechanistically, this effect is mediated by lactate-induced upregulation of serum- and glucocorticoid-inducible kinase 1 (SGK1), which drives ROS accumulation [111].

Thus, targeting the lactate–MDSC axis represents a promising strategy to restore antitumor immunity and improve treatment efficacy.

### Cytotoxic T lymphocytes (CTLs)

A growing body of evidence demonstrates that lactate profoundly influences T cell metabolism, survival, and function, contributing to immune suppression within the tumor microenvironment.

Enhanced lactate production driven by tumor-intrinsic LDHA not only fuels tumor growth but also imposes a profound suppressive effect on antitumor immunity. Elevated lactate accumulation dampens the activity of both T cells and NK cells by suppressing NFAT signaling, thereby reducing IFN-γ production and facilitating immune evasion [112].

Beyond its production, lactate intracellular engagement directly disrupts T cell metabolism. Cytotoxic CD8⁺ T cells rely on pyruvate carboxylase (PC) to convert pyruvate into oxaloacetate, replenishing TCA cycle intermediates such as succinate that are essential for sustaining cytotoxic function. However, excess lactate in the TME diverts pyruvate metabolism toward pyruvate dehydrogenase (PDH)-mediated entry into the TCA cycle, thereby impairing T cell effector activity—a defect that can be reversed by PDH inhibition [113]. In addition, lactate interferes with glucose utilization in CD8⁺ T cells, further compromising their energy supply. Mechanistically, lactate binds to the intracellular domain of glucose transporter 10 (GLUT10), limiting glucose uptake and attenuating cytotoxic responses [114].

Moreover, lactate critically shapes the efficacy of ICB by modulating CD8⁺ T cell survival, infiltration, and effector function within the tumor microenvironment. Specifically, lactate increases the vulnerability of cytotoxic CD8⁺ T cells to activation-induced cell death (AICD), leading to their depletion in KRAS-mutant colorectal cancer. Inhibition of lactate production enhances CD8⁺ T cell infiltration and augments the therapeutic efficacy of ICB in KRAS-mutant MC38 tumor models [115]. Consistently, in melanoma, overexpression of MCT4 markedly increases lactate accumulation in the TME, limiting CD8⁺ T cell infiltration and impairing responses to anti-PD-1 therapy [116]. In hypoxic tumor environments, exhausted T cells markedly upregulate the lactate transporter SLC16A11 (MCT11), leading to increased lactate uptake. Genetic ablation of MCT11 in T cells enhances their cytotoxic function and synergizes with anti-PD-1 therapy [117].

Interestingly, some studies suggest that lactate may also have a beneficial effect on T cells under specific conditions. Pre-treatment of CD8⁺ T cells with lactate in vitro enhances their stemness and anti-tumor capacity upon adoptive transfer, significantly suppressing tumor growth in murine models [118]. These findings highlight the dual, context-dependent roles of lactate in regulating CD8⁺ T cell biology. Further investigation is warranted to dissect the intricate mechanisms through which lactate modulates antitumor immunity and to evaluate therapeutic strategies aimed at targeting lactate metabolism.

Overall, the effects of lactate are pleiotropic and highly context-dependent. Elucidating the key molecular mediators and signaling networks involved in lactate’s actions may provide novel avenues for therapeutic intervention in cancer.

### Regulatory mechanisms and dynamic changes of lactylation in tumor progression

Lactylation is a newly identified post-translational modification that has emerged as a focal point of growing scientific interest. The substantial lactate production resulting from enhanced glycolysis in tumor cells provides an abundant substrate for lactylation. Similar to other post-translational modifications such as acetylation, the occurrence and regulation of lactylation depends on the participation of specific “writer” and “eraser” enzymes, thereby achieving dynamic regulation of cell function and gene expression.

The acetyltransferase e1A binding protein p300 (EP300) and other lysine acetyltransferases (KATs) has been identified as a potential histone lysine lactylation (Kla) “writer” while histone deacetylases (HDAC1-3) and sirtuins (SIRT1-3) function as histone lysine “eraser” or delactylases [119]. Additionally, alanyl-tRNA synthetase 1 (AARS1) acts as an intracellular lactate receptor and a lactyltransferase responsible for global lysine lactylation in tumor cells. Notably, AARS1 targets key oncogenic regulators such as p53 and the YAP1-TEAD complex for lactylation [120, 121]. Mechanistically, AARS1 binds lactate to catalyze the formation of lactate-AMP, subsequently transferring the lactyl group to lysine residues [121]. Other identified lactylation writers include tat-interactive protein, 60 kDa (TIP60), which catalyzes lactylation of vacuolar protein sorting 34 (VPS34) at lysine residues K356 and K781, with TIP60 knockdown reducing VPS34 lactylation [122]. Histone acetyltransferase binding to ORC1 (HBO1) has also been characterized as a lysine lactyltransferase, enriched at transcription start sites, and promotes tumorigenesis through H3K9 lactylation and regulation of oncogene expression [123].

These lactylation writers and erasers participate in regulating tumor progression by mediating lactylation modifications of histones and non-histones. For instance, in intrahepatic cholangiocarcinoma (iCCA), 324 lactylated proteins have been identified, highlighting the widespread nature of this modification. Among them, nucleolin, an RNA-binding protein, undergoes lactylation at lysine 477 in a p300-dependent manner, thereby fostering tumor growth and metastasis through activation of the MAPK signaling pathway [124]. In hepatocellular carcinoma, SIRT3-a known deacetylase-has been shown to mediate delactylation. Specifically, SIRT3 removes lactylation from cyclin E2 (CCNE2) at K348, promoting apoptosis and suppressing tumor cell proliferation [125]. Despite these advances, the molecular mechanisms underlying lactylation remain incompletely understood. Further investigation into the regulatory networks and biological consequences of lactylation is a key research frontier in tumor biology.

### Lactylation coordinates tumor and immune cell crosstalk in the tumor microenvironment

**Fig. 4:**
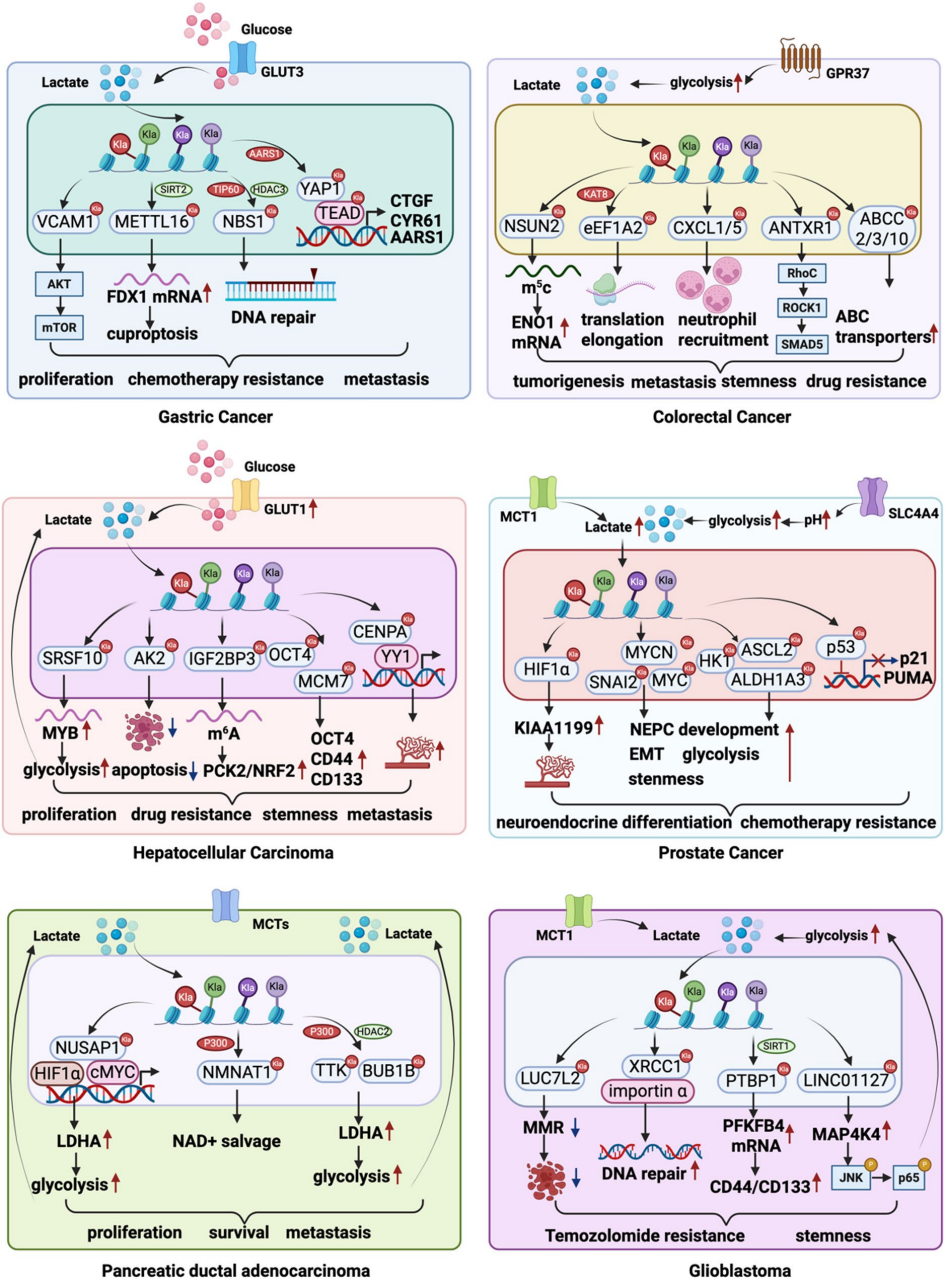
Lactylation drives malignant phenotypes in tumor cells. Lactylation enhances proliferation, increases metastatic potential, maintains stemness, and confers resistance to chemotherapy

Lactylation has been observed across a broad spectrum of malignancies and is implicated in multiple dimensions of tumor biology, including proliferation, metastasis, maintenance of stemness, immune escape, and therapeutic resistance (Fig. 4). Elevated levels of protein lactylation are positively associated with advanced TNM stage, reduced overall survival, and unfavorable clinical outcomes in cancers such as HCC, NSCLC, GC, CRC, PDAC, ocular melanoma, and clear cell renal cell carcinoma [14, 15, 126–130] (Table 1). Furthermore, increased pan-lysine lactylation (pan-Kla) expression correlates with poor immunotherapeutic response in patients with head and neck squamous cell carcinoma (HNSCC) [131], underscoring a potential immunosuppressive role of lactylation.

**Table 1:** Targets and functions of lactylation modifications in various tumors

Cancer type	target	sites	function	Reference
Gastric cancer	NBS1	K388	enhances homologous recombination-mediated DNA repair, contributes to chemotherapy resistance	[134]
	YAP-TEAD1	K108/90	form a positive feedback loop, and facitilates GC proliferation	[120]
	METTL16	K229	results in elevated FDX1 expression, enhances copper death of GC cells	[135]
Colorectal cancer	eEF1A2	K408	contributes to protein synthesis involved in CRC tumorigenesis	[127]
	NSUN2	H3K18	enhances CRC tumorigenesis and metastsis	[136]
	GPR37	H3K18	activates Hippo pathway and enhances glycosis	[137]
	ANTXR1	K453	enhances cancer stemness and drives oxaliplatin resistance	[138]
	ABCC2/3/10	H4K12	leads to chemotherapy resistance	[139]
	RUBCNL	H3K18	promotes CRC cells proliferation and survival in hypoxia	[140]
Hepatocellular carcinoma	AK2	K28	fosters the proliferation and metastasis of HCC cells	[14]
	SRSF10	H3K18	forms the SRSF10/MYB/glycolysis positive feedback loop	[144]
	SKA2/KRT17	H3K56	drives oncogenes expression and facilitates tumor progression	[145]
	CENPA	K124	promotes HCC cell proliferation and tumor growth	[146]
	OCT4	H3K56	maintain the stemness of LCSCs	[148]
	ALDOA	K230/322	enhances proliferation ability and glycosis levels of LCSCs	[148]
	MCM7	H3K18	increases the expression of CSC-related genes and maintain stemness	[149]
	IGF2BP3	K76	drives Lenvatinib resistance	[147]
Prostate cancer	HIF1α	/	upregulates KIAA1199 expression to facilitate angiogenesis and vasculogenic mimicry	[151]
	MYCN/ASCL2/HK1/ALDH1A3	pan-Kla	leads to transcriptional surges of neuroendocrine genes	[154]
	P53	/	promotes enzalutamide resistance and accelerates the malignant progression of prostate cancer	[152]
	MYCN/MYC/Twist2/LDHA	H3K18	reconfigures chromatin accessibility and promotes lineage plasticity, facilitating the emergence of neuroendocrine prostate cancer	[153]
Pancreatic ductal adenocarcinoma	NUSAP1	K34	suppress NUSAP1 degradation and promote PDAC metastasis by forming NUSAP1-LDHA-glycolysis-lactate feedback loop	[157]
	NMNAT1	K128	reinforcing NAD⁺ biosynthesis at the nuclear level and supporting PDAC tumorigenesis	[158]
	TTK/BUB1B	H3K18	promotes PDAC proliferation and migration	[130]
Glioblastoma	LUC7L2	H3K9	inhibits mismatch repair and leads to temozolomide resistance	[161]
	XRCC1	K247	contributes to GBM resistance to radiotherapy and chemotherapy	[162]
	PTBP1	K436	promotes glioma stem cell maintenance	[163]
	LINC01127	H3K18	enhances self-renewal of GBM cells	[164]
Bladder cancer	LCN2	H3K18	promotes bladder cancer cells proliferation, colony formation and migration	[167]
	YY1/YBX1	H3K18	enhances cisplatin resistance	[168]
Cervical cancer	DCBLD1	K127	inhibits DCBLD1 degration and activates pentose phosphate pathway, and enhances cervical cancer progression	[193]
	G6PD	K45	enhances G6PD enzyme activity and promotes tumor proliferation	[194]
Breast cancer	ZWINT/ECT2/ANLN/Ezrin/LDHA	H3K18	initiates a reinforcing feedback axis that supports tumor expansion and metastatic progression in breast cancer	[195]
Ocular melanoma	YTHDF2	H3K18	enhances tumorigenesis by promoting PER1 and TP53 degration	[128]
Non-small cell lung cancer	POM121	H3K18	activatites the POM121/MYC/PD-L1 pathway and promotes immune escape of NSCLC	[15]
	CCNB1	H4K12	promotes acquired pemetrexed resistance of lung cancer brain metastasis by enhancing DNA replication and cell cycle	[196]

**Fig. 5:**
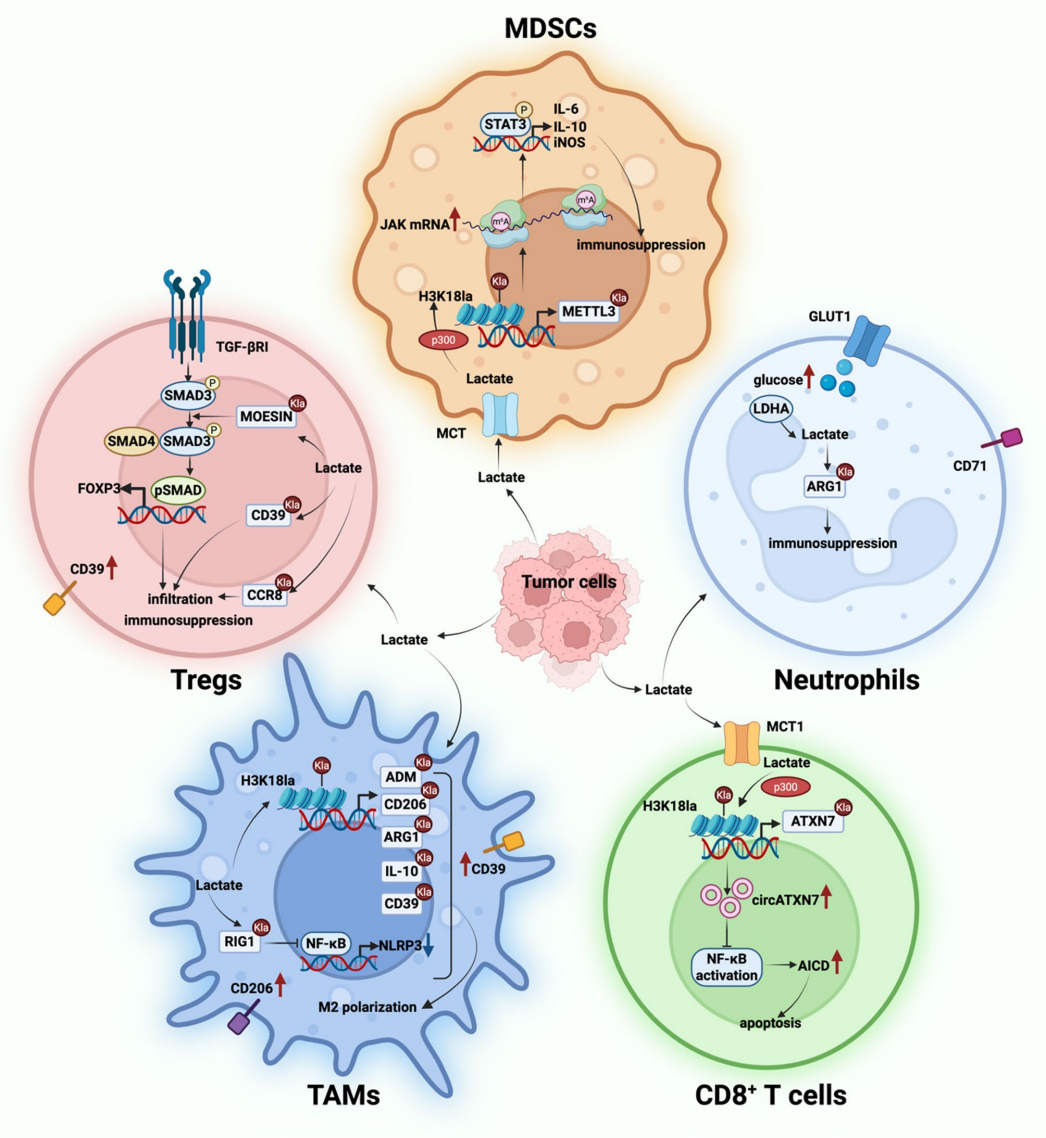
The role of lactylation in immune cells within the TME. Lactylation contributes to the formation of an immunosuppressive TME and serves as a key mechanism underlying immune evasion

Importantly, lactylation is not restricted to tumor cells but also occurs in immune cell subsets within the TME, including Tregs, cytotoxic T cells, TAMs, neutrophils, and MDSCs. By reprogramming transcriptional landscapes and cellular states, lactylation shapes immune responses and contributes to tumor-promoting immunoregulation (Fig. 5). Together, these findings establish lactylation as a central node linking tumor progression with immune suppression in the TME.

### Gastric cancer

In gastric cancer, protein lactylation has emerged as a key regulator of tumor progression. By modulating the expression of downstream targets such as vascular cell adhesion molecule 1 (VCAM1), lactylation drives tumor cell proliferation, EMT, and metastasis [132, 133]. Beyond these oncogenic functions, AARS1 has been identified as a lactyltransferase that catalyzes protein lactylation and promotes the activation of the YAP1–TEAD1 complex. Importantly, AARS1 is itself a transcriptional target of YAP1–TEAD1, thereby forming a positive feedback loop that further amplifies proliferative signaling in gastric cancer [120].

In addition to fueling proliferation and metastasis, lactylation also contributes to therapeutic resistance. Global Kla levels are significantly elevated in chemoresistant gastric cancer tissues and cell lines. Mechanistically, lactate-driven lactylation of nijmegen breakage syndrome 1 (NBS1) at lysine 388 promotes homologous recombination (HR)-mediated DNA repair, thereby contributing to the development of chemoresistance. TIP60 and HDAC3 have been identified as the lactylation “writer” and “eraser,” respectively, modulating the dynamic regulation of NBS1 lactylation [134].

Interestingly, lactylation can also modulate alternative cell fate programs such as copper-induced cell death (cuproptosis). Specifically, lactylation of methyltransferase-like protein 16 (METTL16) at lysine 229 induces m6A modification of ferredoxin 1 (FDX1) mRNA, leading to elevated FDX1 expression and enhanced cuproptosis in gastric cancer cells [135].

### Colorectal cancer

In CRC, KAT8, a well-characterized lysine acetyltransferase, has been identified as a pan-Kla writer. KAT8 specifically mediates lactylation of elongation factor 1 alpha 2 (eEF1A2) at lysine 408, promoting protein synthesis that supports CRC progression. Depletion of KAT8 significantly reduces Kla levels and suppresses CRC cell proliferation in the high-lactate tumor microenvironment [127]. Another study has shown that histone H3 lysine 18 lactylation (H3K18la) activates the transcription of NOP2/Sun RNA methyltransferase 2 (NSUN2) by binding to its promoter, thereby enhancing tumorigenesis and metastasis through the NSUN2/Y-box binding protein 1 (YBX1)/m^5^C-Enolase 1 (ENO1) positive feedback loop [136]. Furthermore, H3K18la is implicated in promoting CRC liver metastasis. The orphan G protein-coupled receptor 37 (GPR37), overexpressed in CRC liver metastases, activates the Hippo pathway and enhances glycolysis, ultimately increasing H3K18la levels and promoting the expression of C-X-C motif chemokine ligand 1 and 5 (CXCL1 and CXCL5) [137].

Beyond its role in proliferation and metastasis, lactylation also integrates metabolic crosstalk with cancer stemness and therapeutic resistance. CAFs-derived lactate is imported into tumor cells via MCT1 and induces lactylation of anthrax toxin receptor 1 (ANTXR1) at K453, which stabilizes ANTXR1 and activates the RhoC/ROCK1/SMAD5 pathway, thereby enhancing cancer stemness and driving oxaliplatin resistance [138]. Besides non-histone targets, histone lactylation further contributes to therapy resistance. Structural maintenance of chromosomes 4 (SMC4) regulates diapause-like cancer cells (DLCCs)-a reversible, quiescent state associated with low proliferative activity and chemoresistance. DLCCs upregulate the transcription of ATP binding cassette (ABC) transporters via increased H4K12 lactylation [139]. Moreover, patients with bevacizumab-resistant CRC exhibit elevated levels of histone lactylation. Inhibition of histone lactylation under hypoxic conditions reduces tumorigenesis, progression, and survival, while enhancing the sensitivity of CRC to bevacizumab treatment [140].

These findings underscore the multifaceted role of lactylation in CRC, extending beyond its function in tumor-intrinsic proliferation, metastasis, stemness, and therapy resistance. Importantly, accumulating evidence highlights that lactylation is not confined to cancer cells, but also profoundly reshapes the tumor immune microenvironment. By modulating the epigenetic and functional states of immune cells such as MDSCs and CTLs, lactylation further promotes immunosuppression and therapeutic resistance, thereby amplifying its oncogenic impact in CRC. Tumor-infiltrating MDSCs show markedly increased levels of methyltransferase-like 3 (METTL3), which enhances their immunosuppressive function via the m6A/JAK1/STAT3 signaling axis. Meanwhile, H3K18la levels are significantly higher in tumor-infiltrating MDSCs compared to Gr1^+^ myeloid cells from the bone marrow and spleen of tumor-free mice. The elevated H3K18la promotes METTL3 expression, with p300 identified as the responsible writer [141]. In KRAS-mutant CRC, CTLs exhibit significantly elevated levels of global histone lactylation compared to those in KRAS wild-type tumors. The lactylation writer EP300 binds to the promoter region of ataxin 7 (ATXN7), leading to increased enrichment of H3K18la at this locus. This modification promotes the transcription of circATXN7, which subsequently enhances CTLs sensitivity to activation-induced cell death [142]. Similarly, in colorectal cancer liver metastasis (CRLM), lactylation of retinoic acid-inducible gene I (RIG-I) at lysine 852 promotes M2 polarization by inhibiting NF-κB signaling and downregulating NLRP3 expression. Blocking RIG-I lactylation effectively suppresses M2 polarization and enhances the sensitivity of CRLM to 5-fluorouracil (5-Fu) [143].

### Hepatocellular carcinoma

In HCC, a comprehensive lactylome–proteome analysis of tumor and adjacent liver tissues from 52 patients with HBV-related HCC identified 9,275 Kla sites, predominantly mapped to non-histone proteins and largely involved in the regulation of metabolic enzymes [14]. Functionally, lactylation has been shown to drive HCC proliferation and metastasis. For example, lactylation of adenylate kinase 2 (AK2) enhances tumor cell growth and dissemination [14]. Histone lactylation also activates oncogenic transcriptional programs: lactylation of H3K18 upregulates the serine and arginine rich splicing factor 10 (SRSF10), which forms a positive feedback loop with MYB to promote glycolysis and proliferation [144]. H3K56 lactylation at the promoter of keratin 17 (KRT17) enhances its transcription, thereby augmenting HCC proliferation [145]. And centromere protein A (CENPA) lactylation at lysine 124 induces its self-activation while upregulating downstream targets such as Yin Yang 1 (YY1) and cyclin D1 (CCND1), further amplifying proliferative signaling [146].

Beyond its role in proliferation, lactylation also contributes to therapeutic resistance. In multiple Lenvatinib-resistant HCC models, increased glycolytic efficiency and lactate accumulation leads to lactylation of insulin like growth factor 2 mRNA binding protein 3 (IGF2BP3) at K76 site. More importantly, elevated expression of IGF2BP3lac is positively correlated with poor prognosis and reduced Lenvatinib responsiveness in patients with HCC. Lactylated IGF2BP3 reprograms serine metabolism by upregulating phosphoenolpyruvate carboxykinase 2 (PCK2), and finally leading to Lenvatinib resistance [147].

In addition, lactylation sustains stemness programs in liver cancer stem cells (LCSCs). Increased H3K56 lactylation at the promoter of octamer-binding transcription factor 4 (OCT4) activates its expression, while non-histone lactylation of aldolase A (ALDOA) at lysine 230/322 enhances the maintenance of LCSC stemness [148]. In addition, in HCC cells, H3K18la upregulates minichromosome maintenance complex component 7 (MCM7) expression, which in turn increases the levels of stemness-associated markers including OCT4, CD44, and CD133, thereby enhancing spheroid-forming capacity [149]. Together, these findings highlight lactylation as a multifaceted epigenetic mechanism that sustains stemness in HCC, underscoring its role in tumor plasticity and progression.

Beyond tumor-intrinsic effects, lactylation also acts at the interface of tumor and immune compartments, shaping the immunological landscape of HCC. In patients with HCC, the level of membrane-organizing extension spike protein (MOESIN) lactylation in Tregs has been found to correlate with immunotherapy responsiveness. Specifically, lactylation of MOESIN at lysine 72 enhances TGF-β signaling in Tregs through TGF-β receptor I (TGF-βRI) [150]. Moreover, SRSF10 facilitates tumor progression by promoting M2 macrophage polarization, thereby reducing CD8⁺ T cells infiltration and fostering an immunosuppressive tumor microenvironment. Mechanistically, SRSF10 enhances lactate production in HCC cells, which subsequently elevates H3K18 lactylation levels in M2 macrophages. This histone modification upregulates the expression of pro-tumorigenic genes including IL-10, CD206, adrenomedullin (ADM), and ARG1, amplifying the immunosuppressive function of M2 macrophages [144].

### Prostate cancer

In prostate cancer (PCa), lactylation contributes to multiple malignant phenotypes, including metastasis, therapy resistance, and neuroendocrine differentiation. Elevated intracellular lactate transported via MCT1 promotes the lactylation of HIF1α, which transcriptionally upregulates cell migration–inducing hyaluronidase 1 (CEMIP, also known as KIAA1199). CEMIP enhances angiogenesis and vasculogenic mimicry by increasing VEGFA secretion, thereby facilitating tumor dissemination [151].

Lactylation also plays a role in drug resistance. Treatment with the androgen receptor signaling inhibitor enzalutamide (Enz) increases P53 lactylation in PCa cells. This modification represses the expression of P53 target genes, whereas downregulation of the transporter SLC4A4 reduces P53 lactylation and restores P53-dependent transcription, partially reversing Enz resistance [152].

Furthermore, lactylation regulates cellular plasticity and neuroendocrine prostate cancer (NEPC) transformation. Overexpression of ZEB1 drives global pan-Kla and H3K18la, particularly at ZEB1 target loci enriched for neurogenesis, EMT, stemness, and glycolysis pathways. This lactylation-dependent transcriptional reprogramming facilitates NEPC progression [153]. Conversely, The proteins NUMB endocytic adaptor protein (NUMB) and NUMB like endocytic adaptor protein (NUMBL) function as negative regulators by suppressing lactylation at the promoters of oncogenic drivers such as MYCN proto-oncogene (MYCN), achaete-scute family BHLH transcription factor 2 (ASCL2), hexokinase 1 (HK1), and aldehyde dehydrogenase 1 family member A3 (ALDH1A3), thereby constraining neuroendocrine transdifferentiation [154].

Apart from regulating tumor cells, lactylation extends its impact to the immune milieu of PCa. In a mouse model of prostate cancer, combined inhibition of MEK and PI3K pathways reduces lactate levels in the TME and diminishes H3K18la in TAMs, resulting in an 80% overall response rate. However, in the remaining 20% of non-responders, reactivation of the Wnt/β-catenin pathway restores lactate secretion and H3K18la levels in TAMs, contributing to treatment resistance [155, 156].

### Pancreatic ductal adenocarcinoma

Elevated levels of pan-Kla and H3K18la have been observed in PDAC tissues, with high H3K18la expression significantly correlated with advanced American Joint Committee on Cancer (AJCC) stage and poor prognosis. Histone lactylation has been shown to promote PDAC cell proliferation and migration both in vitro and in vivo. Mechanistically, EP300 and HDAC2 have been identified as the histone lactylation “writer” and “eraser,” respectively. And several downstream targets, such as NUSAP1, TTK protein kinase (TTK), BUB1 mitotic checkpoint serine/threonine kinase B (BUBIB), and nicotinamide nucleotide adenylyltransferase 1 (NMNAT1), are found to be regulated by histone lactylation and thus involved in the regulation of PDAC metabolism, proliferation, and metastasis [130, 157, 158].

In addition to proliferation, lactylation is also crucial for maintaining CSCs properties in PDAC. Lactate generated through SIRT4-mediated glycolysis fuels histone lactylation at H3K9 and H3K18, which in turn activates stemness-associated transcriptional programs. Notably, this modification also induces PD-L1 expression and activates the PD-1 checkpoint pathway, highlighting a dual role in sustaining stemness and enabling immune evasion [159].

Consistent with its role in shaping the immune landscape, histone lactylation levels are further elevated in PDAC liver metastases compared with primary tumors. These epigenetic changes are tightly associated with the establishment of an immunosuppressive microenvironment, underscoring the multifaceted contribution of lactylation to PDAC progression [160].

### Glioblastoma

In glioblastoma (GBM), lactylation is essential for mediating drug resistance and sustaining cancer stemness, through transcriptional control of downstream effectors [161–164].

Temozolomide (TMZ), a widely used oral chemotherapeutic agent and first-line treatment for GBM, is primarily effective during early disease stages but often leads to resistance with prolonged use. Elevated levels of global lactylation and H3K9 lactylation (H3K9la) have been observed in TMZ-resistant GBM cells, with chronic TMZ exposure further increasing intracellular H3K9la levels. Mechanistically, H3K9la is enriched at the promoter of LUC7 like 2, pre-mRNA splicing factor (LUC7L2), enhancing its transcription and expression. This upregulation suppresses MutL homolog 1 (MLH1), a key mismatch repair gene, thereby contributing to TMZ resistance [161]. A similar mechanism is described in another study, where ALDH1A3-driven activation of pyruvate kinase leads to lactate accumulation, enhancing lactylation at lysine 247 of X-Ray repair cross complementing 1 (XRCC1). This modification promotes resistance to both radiotherapy and chemotherapy in GBM [162].

Compared with differentiated glioma cells, glioma stem cells (GSCs) exhibit markedly elevated global lactylation, which is essential for maintaining their stem-like properties. Mechanistically, lactylation of polypyrimidine tract–binding protein 1 (PTBP1) at K436 enhances the stability of 6-phosphofructo-2-kinase/fructose-2,6-bisphosphatase 4 (PFKFB4) mRNA, a glycolytic rate-limiting enzyme, thereby promoting glycolysis and sustaining stemness. This modification is dynamically regulated, as SIRT1 functions as a delactylase for PTBP1 [163]. In parallel, H3K18la has been shown to reinforce GSC stemness by upregulating the long noncoding RNA LINC01127 [164]. Together, these findings highlight lactylation as a key metabolic–epigenetic mechanism that sustains GSC stemness and drives glioma progression.

Apart from their tumor-intrinsic roles in resistance and stemness, lactylation-dependent mechanisms in GBM extend to the immune microenvironment, where they reprogram regulatory and innate immune cells to reinforce immunosuppression and therapeutic resistance. In a murine GBM model, lactate induces H3K18 lactylation, which promotes the transcriptional activity of CD39 and C-C motif chemokine receptor 8 (CCR8) in Tregs. Notably, treatment with the lactate production inhibitor oxamate reduces tumor-infiltrating Treg cells by suppressing CCR8 expression, thereby enhancing CAR-T cell activation within the tumor microenvironment [165]. Moreover, elevated lactate levels promote lactylation, which upregulates ARG1 expression in neutrophils under hypoxic conditions. This in turn impairs the cytotoxic function of CD8⁺ T cells, contributing to immune evasion and tumor progression [166].

### Bladder cancer

In bladder cancer (BCa), both global lysine lactylation and H3K18la levels are significantly upregulated. Functionally, H3K18la facilitates tumor growth via enhanced expression of lipocalin 2 (LCN2). Conversely, circXRN2 has been shown to attenuate both global and H3K18-specific lactylation by modulating Hippo signaling, thereby suppressing bladder cancer proliferation and metastasis [167]. Notably, increased H3K18la levels are also detected in cisplatin-resistant BCa cell lines [168]. Mechanistically, H3K18la enhances the transcription of oncogenic regulators YY1 and YBX1, thereby facilitating the development of cisplatin resistance in bladder cancer.

### Targeting lactate metabolism and lactylation provides novel therapeutic opportunities

As discussed above, lactate and lactylation modifications serve as critical regulators of tumor progression. Consequently, targeting lactate metabolism and lactylation have gained attention for their capacity to reshape the immunosuppressive tumor microenvironment and impede cancer development, holding significant potential for clinical translation. Current therapeutic approaches to targeting lactate metabolism include four main strategies: (1) inhibiting lactate production, (2) blocking lactate transport, (3) antagonizing lactate receptors, and (4) directly eliminating lactate within the tumor microenvironment (Table 2).

### Suppression of lactate production

LDH is a tetrameric enzyme composed of LDHA and/or LDHB subunits, whose composition varies by tissue type. LDHA is predominantly expressed in skeletal muscle and liver, where it catalyzes the conversion of pyruvate to lactate. In contrast, LDHB is mainly found in the heart, kidneys, and brain, facilitating the reverse reaction—from lactate to pyruvate. Among these, LDHA-mediated lactate accumulation represents a major source of tumor-derived lactate. Elevated expression of LDHA is commonly correlated with unfavorable prognosis across multiple cancer types. Genetic silencing of LDHA has been shown to suppress glycolysis and reduce lactate production across various tumor types, thereby attenuating tumor growth. Accordingly, LDHA is recognized as a promising therapeutic target in oncology. Several small-molecule LDHA inhibitors have been developed and shown to exert antitumor effects [169, 170].

Oxamate, a structural analog and isostere of pyruvate, competes with pyruvate at the LDH catalytic site, forming an inactive enzyme-substrate complex and thus effectively inhibiting LDH activity [171]. Specifically, MET transcriptional regulator MACC1 promotes glycolysis and lactate production via the PI3K/AKT pathway, contributing to resistance against trastuzumab in gastric cancer. Combined treatment with oxamate and trastuzumab significantly inhibits MACC1-driven tumor growth and glycolysis in xenograft models [172]. Similarly, oxamate reduces lactate levels in NSCLC cells and enhances their radiosensitivity [173].

FX11 is also a commonly used small molecule inhibitor of LDHA [174]. In PDAC, lactate secreted by tumor cells stimulates IL-6 production from cancer-associated fibroblasts. This lactate-IL-6 axis fosters an immunosuppressive tumor microenvironment and facilitates PDAC progression. Silencing LDHA or treatment with the LDHA inhibitor FX11 suppresses tumor growth in CAFs-rich PDAC models [76]. In prostate cancer, FX11 treatment mitigates lactate-mediated angiogenesis and tumor cell migration [52], and similarly, FX11 inhibits LDHA-dependent cell proliferation, migration, and invasion in papillary thyroid carcinoma both in vitro and in vivo [175].

Beyond LDH, other glycolytic enzymes are also involved in lactate biosynthesis. Thus, inhibition of glycolysis can indirectly reduce lactate production. For instance, elevated lactylation of IGF2BP3 contributes to Lenvatinib resistance in HCC. In an orthotopic HCC mouse model, treatment with the glycolytic inhibitor 2-deoxy-D-glucose (2-DG) downregulated IGF2BP3 expression and improved sensitivity to Lenvatinib [147].

### Inhibition of lactate transport

Among the monocarboxylate transporters MCT1-4, MCT1 and MCT4 have been most extensively studied in cancer. MCT1 primarily mediates lactate influx, while MCT4 is mainly responsible for lactate efflux. Together, these transporters are essential for mediating metabolic crosstalk both among tumor cells and between tumor cells and stromal or immune components within the tumor microenvironment. Their elevated expression in various malignancies has positioned MCT1 and MCT4 as promising therapeutic targets in cancer treatment [26].

In metastatic CRC, resistance to first-line anti-epidermal growth factor receptor (EGFR) therapy can be reversed by inhibiting MCT1. Cetuximab-resistant CRC cells with KRAS mutations display heightened glycolytic activity and rely on lactate as an alternative energy source to sustain proliferation. Treatment with the MCT1 inhibitor AR-C155858 effectively blocks lactate uptake and oxidation, resulting in suppressed tumor growth [176]. Similarly, in tamoxifen-resistant MCF-7 breast cancer cells, AR-C155858 reduces proliferation, migration, and survival [177]. Another MCT1 inhibitor, AZD3965, has demonstrated antitumor activity in xenograft models by suppressing tumor growth, enhancing immune cell infiltration and cytotoxicity, and increasing tumor radiosensitivity [178, 179]. Beyond its direct antitumor effects, AZD3965 also mitigates lactate-driven immunosuppression. In multiple myeloma, patients exhibit significantly elevated circulating lactate levels compared to healthy individuals, which promotes the expansion of MDSCs and Tregs. Treatment with AZD3965 effectively reduces these immunosuppressive populations, thereby restoring antitumor immunity [180]. Consistent with these preclinical findings, a Phase I clinical trial (NCT01791595) assessing the safety and maximum tolerated dose of AZD3965 in advanced tumors has demonstrated that clinically relevant concentrations can be achieved with acceptable tolerability [181]. These findings underscore its translational potential as both a metabolic and immunomodulatory therapeutic strategy.

In contrast, MCT4 inhibition offers a strategic route to counteract lactate-driven immunosuppression, thereby enhancing the efficacy of ICB. In a 3D human CRC spheroid model co-cultured with peripheral leukocytes, MCT4 inhibition alone restored T-cell activity. When combined with anti-PD-L1 or anti-PD-1 therapy, it significantly boosted immune cell infiltration and impaired tumor spheroid viability. These findings were mirrored in vivo using an MC38 mouse model, where dual inhibition of MCT4 and PD-L1 or PD-1 elevated intratumoral pH, increased T cells infiltration, augmented cytotoxicity, and ultimately prolonged survival [116, 182]. In HCC, high MCT4 expression correlates with poor prognosis and serves as a potential biomarker for anti-PD-1 therapy responsiveness. Notably, MCT4 inhibition with VB124 activates the ROS/NF-κB signaling pathway, leading to increased expression of CXCL9 and CXCL10, which promotes CD8⁺ T cell infiltration into the tumor microenvironment. Thus, targeting MCT4 represents a compelling strategy to enhance the therapeutic efficacy of ICB in HCC [183].

### Targeting lactate receptor

Lactate functions as an agonist of the G protein-coupled receptor HCAR1 (also known as GPR81), exerting its effects through both autocrine and paracrine signaling. Activation of GPR81 has been shown to promote angiogenesis, immune evasion, and resistance to therapy. Therefore, targeting lactate receptors represents a promising strategy to reverse the immunosuppressive tumor microenvironment, suppress tumor growth, enhance antitumor immune responses, and ultimately inhibit cancer progression [29]. In CRC, HCAR1 signaling promotes the secretion of chemokines CCL2 and CCL7 by tumor cells, which in turn recruit CCR2^+^ PMN-MDSCs. These cells inhibit CD8⁺ T cell activation, thereby facilitating immune escape and increasing tumor burden. In a murine CRC model, the antihypertensive agent reserpine is shown to block lactate-induced HCAR1 signaling, suppress the recruitment of CCR2 + PMN-MDSCs, restore CD8⁺ T cell-mediated antitumor immunity, and enhance tumor sensitivity to anti-PD-1 therapy [107].

### Direct removal of lactate from tumors

Lactate oxidase (LOX), an 80 kDa natural enzyme, catalyzes the conversion of lactate to pyruvate and hydrogen peroxide (H_2_O_2_) in vivo, thereby effectively reducing lactate levels. As a result, LOX has attracted significant attention in cancer therapy. The use of nanomaterials or other emerging delivery systems to transport LOX to targeted sites has shown promising anti-tumor effects. Nanosystems carrying LOX can inhibit various pro-tumor effects mediated by lactate, including anti-angiogenesis, enhancing chemotherapy sensitivity, and boosting immune responses [184, 185]. For example, a metal-organic framework (MOF) system that encapsulates both LOX and a signal-regulatory protein α (SIRPα) genome editing plasmid exhibits a synergistic effect. This system depletes lactate and blocks SIRPα signaling, which enhances TAMs phagocytosis and repolarizes TAMs to the M1 phenotype, ultimately inhibiting tumor growth [186]. However, the H_2_O_2_ produced during the LOX reaction is a key factor limiting the clinical application of LOX-carrying nanomaterials due to potential toxicity.

In addition to LOX, the LDH inhibitors can also be delivered via nanosystems. For instance, Galloflavin, a natural polyphenol and LDH inhibitor, reduces lactate production by inhibiting the conversion of pyruvate to lactate. When incorporated into nanosystems using metal-phenol coordination, Galloflavin alleviates the acid-suppressive immune microenvironment of breast cancer. Furthermore, nanocomplexes of Galloflavin combined with photosensitizers can induce immune cell death and activate the immune system, thereby inhibiting tumor growth [187]. Although this delivery system offers novel therapeutic strategies, its safety and efficacy still require further validation.

### Targeting lactylation

Lactate acts as a primary metabolic substrate for protein lactylation, and inhibition of lactate production is anticipated to diminish intracellular lactylation levels [188]. Notably, lactylation of NBS1 enhances DNA damage repair and promotes chemoresistance, contributing to poor prognosis in cancer patients, with LDHA playing a central enzymatic role in this process. Stiripentol, an FDA-approved drug for refractory epilepsy, is a well-characterized LDHA inhibitor. In gastric cancer cells, stiripentol suppresses lactate production and inhibits NBS1 K388 lactylation, thereby impairing DNA repair efficiency. Preclinical studies have demonstrated that stiripentol exhibits strong synergistic anti-tumor effects when combined with cisplatin or ionizing radiation in patient-derived organoid and xenograft models [134]. Furthermore, stiripentol can cross the blood–brain barrier and inhibit LDHA activity in glioblastoma cells. Functioning as a lactylation inhibitor, it enhances the sensitivity of GBM cells to TMZ treatment in both in vitro and in vivo models [161].

Other agents, such as oxamate and 2-DG, have also been shown to reduce lactylation levels in endometrial cancer and HCC cells, thereby suppressing lactylation-mediated gene activation and inhibiting tumor proliferation and migration [147, 189]. In a lenvatinib-resistant mouse liver cancer model, treatment with 2-DG not only lowered IGF2BP3 lactylation levels but also restored sensitivity to lenvatinib [147]. Additionally, demethylzeylasteral (DML), a bioactive compound extracted from Tripterygium wilfordii Hook F, has demonstrated anti-tumor efficacy in a nude mouse xenograft model of HCC. Mechanistically, DML reduces lactate production and histone H3 lactylation in liver cancer stem cells, thereby promoting apoptosis and suppressing proliferation and migration [190].

Targeting lactylation in combination with immunotherapy has emerged as a promising strategy to suppress tumor progression and offers novel insights into cancer treatment. In NSCLC, H3K18 lactylation facilitates immune evasion by impairing CD8⁺ T cell function through activation of the POM121 transmembrane nucleoporin (POM121)/MYC/PD-L1 signaling axis. The LDHA inhibitor Oxamate markedly reduces intracellular H3K18la levels and disrupts the recruitment of the lactylation writer EP300 to the promoter region of POM121. Notably, the combination of Oxamate and anti-PD-1 therapy restores CD8⁺ T cell cytotoxicity and significantly inhibits tumor growth [15].

**Table 2:** Drugs targeting lactate metabolism and lactylation

Molecule	Mechanism	Condition	Clinical trial	Reference
Oxamate	inhibits LDH	gastric cancer NSCLC	N/A	[172, 173]
FX11	inhibits LDHA	PDAC, prostate cancer, papillary thyroid carcinoma	N/A	[52, 76, 175]
Stiripentol	inhibits LDHA	gastric cancer, GBM	Approved by FDA for refractory epilepsy	[134, 161]
2-deoxy-D-glucose	inhibit glycolysis	advanced solid tumors	NCT00096707 Phase 1, Completed	[191]
AR-C155858	inhibits MCT1	CRC, breast cancer	N/A	[176, 177]
AZD3965	inhibits MCT1	advanced solid tumors, lymphoma	NCT01791595 Phase 1, Completed	[181]
lonidamine	inhibits MCT4	CRC	N/A	[116]
VB124	inhibits MCT4	HCC	N/A	[183]
Reserpine	inhibits HCAR1	CRC	Approved by FDA for hypertension	[107]

## Conclusion and perspectives

Lactate is not only a byproduct of tumor cell metabolism, but also a signaling molecule that mediates transcellular communication between tumor cells and immune cells. Tumor cells excrete a large amount of lactate into the TME through MCTs, a process coupled with proton efflux that acidifies the TME and impairs the function of effector T cells and NK cells. Concurrently, lactate can be imported by immune cells within the TME, further influencing their metabolic and immunological states. After intracellular accumulation, it regulates gene expression by inducing lactylation, thereby enhancing the function of immunosuppressive cells. In addition, lactate interferes with immune recognition by inducing PD-L1 expression and limiting antigen presentation, further achieving immune escape. These findings suggest that lactate, as a “metabolism-mediated immunomodulator”, plays a bridging role in tumor immunoregulation. Therefore, in-depth research on the signaling mechanism of lactate between tumor cells and immune cells will help develop new metabolic-immune dual-targeting strategies to improve the response rate and durability of tumor immunotherapy.

Lactate is identified as an epigenetic factor and undertakes the important task of providing substrates for lactylation. Lactylation is a novel post-translational modification of protein that occurs on lysine residues of histones or other proteins. By affecting the spatial structure of chromatin, it increases DNA accessibility and thus regulates gene expression. The discovery of lactylation fills the understanding of the physiological effects of lactate and its complex role in tumors [192]. Lactyltransferase involved in lactylation (such as EP300, HBO1) and delactylation enzymes (such as HDAC1-3, SIRT1-3) have been reported, however, other writers and erasers regarding the lactylation still require extensive research to discover.

Lactylation is similar to other epigenetic modifications, regulating gene expression by affecting chromatin accessibility. And many acetylated writers and erasers are also responsible for regulating lactylation. So, whether there is a synergistic or antagonistic effect between lactylation and other epigenetic modifications is a question worth considering. The combined effects of multiple epigenetic modifications make the regulatory mechanism of tumors and tumor microenvironment more complex.

The key role of lactylation in TME has been widely paid attention and recognized. Lactylation occurring in a variety of immune cells have led to suppression of the immune microenvironment and the development of tumor. However, there are still a lot of gaps in genes, sites and detailed mechanisms for lactylation in immune cells. At present, the functions of lactylation in immune cells are mainly concentrated in CD8⁺ T cells, Tregs, MDSCs and TAMs. The role of lactylation in other immune cells such as NK cells, dendritic cells, B cells and their function in tumor progression are worth exploring.

In summary, current studies have confirmed that lactate and its mediated lactylation modification play a key role in tumorigenesis, progression and immune escape. At present, drugs used to block lactate production, such as LDHA inhibitors, and MCT1/4 inhibitors that interfere with lactate output have shown certain anti-tumor potential in some preliminary studies, but their selectivity and toxicity are still difficult to meet clinical requirements. In addition, specific small molecule inhibitors for “writers” and “erasers” in the lactylation modification regulatory mechanism are still in their infancy, lacking systematic screening and functional verification. Therefore, future research needs to focus on the development of new lactate/lactylation inhibitors with clear targets, strong tissue penetration, and low toxic side effects, and combine them with drug delivery systems to improve their enrichment efficiency in tumor tissues. At the same time, combined treatment strategies with immune checkpoint inhibitors or other metabolic regulation drugs should also be explored in depth to enhance the clinical translation potential and efficacy of lactate targeted therapy.

## Data Availability

Not applicable.

## Abbreviations

2-DG: 2-deoxy-D-glucose
AARS1: Alanyl-tRNA synthetase 1
ABC: ATP binding cassette
ADM: Adrenomedullin
AICD: Activation-induced cell death
AJCC: American Joint Committee on Cancer
AK2: Adenylate kinase 2
ALDH1A3: Aldehyde dehydrogenase 1 family member A3
ALDOA: Aldolase A
ALKBH5: AlkB homolog 5, RNA demethylase
ANLN: Anillin actin binding protein
ANTXR1: Anthrax toxin receptor 1
ARG1: Arginase 1
ASCL2: Achaete-scute family BHLH transcription factor 2
ASCT2: Amino acid transporter type 2
ATP: Adenosine triphosphate
ATXN7: Ataxin 7
BDNF: Brain-derived neurotrophic factor
BUB1B: BUB1 mitotic checkpoint serine/threonine kinase B
CAFs: Cancer-associated fibroblasts
cAMP: Cyclic Adenosine monophosphate
CCL2/7/17: C-C motif chemokine ligand 2/7/17
CCND1: Cyclin D1
CCNE2: Cyclin E2
CCR4/8: C-C motif chemokine receptor 4/8
CENPA: Centromere protein A
COX2: Cyclooxygenase-2
CRC: Colorectal cancer
CRLM: Colorectal cancer liver metastasis
CSCs: Cancer stem cells
CTGF: Connective tissue growth factor
CTLA-4: Cytotoxic T-lymphocyte associated protein 4
CTLs: Cytotoxic T lymphocytes
CX3CL1: C-X3-C motif chemokine ligand 1
CXCL1/5/9/10/12: C-X-C motif chemokine ligand 1/5/9/10/12
CYR61: Cysteine rich angiogenic inducer 61
DCBLD1: Discoidin, CUB and LCCL domain containing 1
DCs: Dendritic cells
DKK2: Dickkopf associated protein 2
DLCCs: Diapause-like cancer cells
ECT2: Epithelial cell transforming 2
eEF1A2: Eukaryotic translation elongation factor 1 alpha 2
EGFR: Epidermal growth factor receptor
EMT: Epithelial–mesenchymal transition
ENO1: Enolase 1
EP300: E1A binding protein p300
ESCC: Esophageal squamous cell carcinoma
F-1,6-BP: Fructose-1,6-bisphosphate
F-6-P: Fructose-6-phosphate
FDX1: Ferredoxin 1
FOXQ1/FOXO3/FOXM1/FOXP3: Forkhead box Q1/O3/M1/P3
G-6-P: Glucose-6-phosphate
G6PD: Glucose-6-phosphate dehydrogenase
GC: Gastric cancer
GLS: Glutaminase
GLUD: Glutamate dehydrogenase
GLUT10: Glucose transporter 10
GPR37/65/81/132: G protein-coupled receptor 37/65/81/132
GZMB: Granzyme B
H3K18la: Histone H3 lysine 18 lactylation
HBO1: Histone acetyltransferase binding to ORC1
HCC: Hepatocellular carcinoma
HDAC: Histone deacetylase
HIF1α: Hypoxia-inducible factor 1-alpha
HISLA: HIF-1α-stabilizing long noncoding RNA
HK1/2: Hexokinases 1/2
HMGB1: High mobility group box 1
HNSCC: Head and neck squamous cell carcinoma
HR: Homologous recombination
ICB: Immune checkpoint blockade
iCCA: Intrahepatic cholangiocarcinoma
IFN-α: Interferon-alpha
IFN-γ: Interferon-gamma
IGF2BP3: Insulin like growth factor 2 mRNA binding protein 3
KATs: Lysine acetyltransferases
KIAA1199 (CEMIP): Cell migration inducing hyaluronidase 1
Kla: Lysine lactylation
KRT17: Keratin 17
LCN2: Lipocalin 2
LCSCs: Liver cancer stem cells
LDH: Lactate dehydrogenase
LOX: Lactate oxidase
LRP6: Lipoprotein receptor-related protein 6
LUC7L2: LUC7 like 2, pre-mRNA splicing factor
MACC1: MET transcriptional regulator MACC1
MCTs: Monocarboxylate transporters
MCM7: Minichromosome maintenance complex component 7
MDSCs: Myeloid-derived suppressor cells
ME1: Malic enzyme 1
METTL3/16: Methyltransferase-like protein 3/16
MHCII: Major histocompatibility complex class II
MLH1: MutL homolog 1
MMPs: Matrix metalloproteinases
MOESIN: Membrane-organizing extension spike protein
MOF: Metal-organic framework
mregDCs: Mature regulatory dendritic cells
MYCN: MYCN proto-oncogene
NADPH: Nicotinamide adenine dinucleotide phosphate (Reduced form)
NBS1: Nijmegen breakage syndrome 1
NEPC: Neuroendocrine prostate cancer
NFAT1: Nuclear factor of activated T cells 1
NK: Natural killer
NLRP3: NLR family pyrin domain containing 3
NMNAT1: Nicotinamide nucleotide adenylyltransferase 1
NRF2: Nuclear factor erythroid 2–related factor 2
NRP2: Neuropilin 2
NSCLC: Non-small cell lung cancer
NSG1: Neuronal vesicle trafficking associated 1
NSUN2: NOP2/Sun RNA methyltransferase 2
NUMB: NUMB endocytic adaptor protein
NUMBL: NUMB like endocytic adaptor protein
NUSAP1: Nucleolar and spindle associated protein 1
OCT4: Octamer-binding transcription factor 4
OXPHOS: Oxidative phosphorylation
Pan-Kla: Pan-lysine lactylation
PC: Pyruvate carboxylase
PCK2: Phosphoenolpyruvate carboxykinase 2
PCSK9: Proprotein convertase subtilisin/kexin type 9
PD-1: Programmed death-1
PDAC: Pancreatic ductal adenocarcinoma
pDCs: Plasmacytoid dendritic cells
PDH: Pyruvate dehydrogenase
PD-L1: Programmed death-ligand 1
PEP: Phosphoenolpyruvate
PFKFB4: 6-phosphofructo-2-kinase/fructose-2,6-bisphosphatase 4
PFKL: Phosphofructokinase-1, liver type
PFKM: Phosphofructokinase-1
PGC-1α: peroxisome proliferator-activated receptor-γ coactivator-1α
PKA: Protein kinase A
PKM2: Pyruvate kinase M 2
PMN-MDSCs: Polymorphonuclear myeloid-derived suppressor cells
POM121: POM121 transmembrane nucleoporin
PTBP1: Polypyrimidine tract–binding protein 1
RIG-I: Retinoic acid-inducible gene I
ROS: Reactive oxygen species
RUBCNL: Rubicon like autophagy enhancer
S100A8/9: S100 calcium binding protein A8/9
SETDB1: SET domain bifurcated 1
SGK1: serum- and glucocorticoid-inducible kinase 1
SIRPα: Signal-regulatory protein α
SIRT: Sirtuin
SLC16: Solute carrier family 16
SMC4: Structural maintenance of chromosomes 4
SNAI2: Snail family transcriptional repressor 2
SPI1: Spi-1 proto-oncogene
SREBP2: Sterol regulatory element-binding protein 2
SRSF10: Serine and arginine rich splicing factor 10
STK11: Serine/threonine kinase 11
SUCNR1: Succinate receptor 1
TAMs: Tumor-associated macrophages
TAZ: Transcriptional coactivator with PDZ-binding motif
TAp73: Transactivation domain-containing p73
TCA: Tricarboxylic acid
TEAD: TEA domain family member
TGF-β: Transforming growth factor-beta
TGF-βRI: Transforming growth factor-beta receptor I
TIP60: Tat-interactive protein, 60 kDa
TME: Tumor microenvironment
TMEM105: Transmembrane protein 105
Tregs: Regulatory T cells
TTK: TTK protein kinase
USP39: Ubiquitin-specific peptidase 39
VCAM1: Vascular cell adhesion molecule 1
VEGFA: Vascular endothelial growth factor A
VPS34: Vacuolar protein sorting 34
XRCC1: X-Ray repair cross complementing 1
YBX1: Y-box binding protein 1
YTHDF2: YTH N6-Methyladenosine RNA Binding Protein 2
YY1: Yin Yang 1
ZEB1: Zinc finger E-box-binding homeobox 1
ZWINT: ZW10 interacting kinetochore protein
